# Does Food Habits and Malnutrition Affect Health Perception Among Diabetic Patients? A Mediation and Moderation Analysis

**DOI:** 10.3390/healthcare13101093

**Published:** 2025-05-08

**Authors:** Sufyan Habib, Nawaf N. Hamadneh, Asif Hasan, Ahmed S. M. Almamy, Bandar N. Hamadneh

**Affiliations:** 1 Department of Business Administration, College of Administration and Finance, Saudi Electronic University, Riyadh 11673, Saudi Arabia; s.habib@seu.edu.sa (S.H.); ah.hassan@seu.edu.sa (A.H.); a.almamy@seu.edu.sa (A.S.M.A.); 2 Department of Basic Sciences, College of Science and Theoretical Studies, Saudi Electronic University, Riyadh 11673, Saudi Arabia; 3 Department of Nutrition and Dietetics, College of Agriculture, Ajloun National University, Ajloun 26810, Jordan

**Keywords:** malnutrition, food habits, health perception, diabetic patients, dietary knowledge, dietitian-led interventions

## Abstract

Background: The prevalence of diabetes among young individuals has reached concerning levels, posing significant public health risks and exacerbating the economic burden on healthcare systems. The health outcomes of individuals with diabetes are heavily influenced by malnutrition and unhealthy dietary habits, which not only hamper effective blood glucose management but also negatively affect overall health perceptions. Introduction: This study analyzes the factors influencing malnutrition, food habits, and health perceptions among diabetic patients. Methods: A well-structured questionnaire was designed to collect data. A cross-sectional survey of 503 diabetic patients across various regions in India was conducted. Additionally, structural equation modeling, as well as mediation and moderation analyses, were performed. Results: The study findings revealed that dietary knowledge, dietitian-led interventions, and economic factors significantly influenced malnutrition and health outcomes. Conversely, nutritional quality did not emerge as a significant predictor. Discussions: The study will help pharmaceutical companies, governments, and healthcare practitioners in marketing dietary supplements, design focused dietary programs, and develop health education campaigns to improve diabetes patients’ quality of life. The findings illuminated the critical roles of dietary knowledge, dietitian-led interventions, economic factors, and lifestyle modifications in managing malnutrition and enhancing health outcomes. Conclusions: The study demonstrated significant mediation and moderation effects, emphasizing the complex interplay between food habits and malnutrition on health perceptions. However, nutritional quality was not a significant predictor, and the research underscored the necessity of holistic, personalized interventions. This will also help medical marketers in devising their marketing strategies.

## 1. Introduction

Malnutrition and poor dietary habits significantly influence the health outcomes of diabetic patients, a population that is rapidly increasing in India. Malnutrition among diabetic patients can exacerbate the severity of diabetes by impairing the body’s ability to manage blood glucose levels [1]. Dietary habits, particularly the consumption of nutrient-dense foods, play an important role in treating diabetes and reducing associated problems [2]. Furthermore, health perception, defined as an individual’s awareness and comprehension of their health state, can potentially influence the association between eating behaviors and health outcomes [3]. The worldwide burden of nutrition-related non-communicable illnesses, notably diabetes, is significant. Diabetes, a chronic metabolic disorder marked by elevated blood glucose levels, poses significant global health challenges, including risks to the cardiovascular system, kidneys, nerves, and vision [4]. Type 2 diabetes, prevalent among adults due to insulin resistance or inadequate insulin production, has surged in prevalence over the past three decades across income levels [5]. Conversely, type 1 diabetes results from an autoimmune deficiency in insulin production. Alarmingly, over 830 million individuals worldwide are affected, with most residing in low- and middle-income countries. Despite the rising prevalence, more than 50% remain untreated, exacerbating health inequities [4]. Unfortunately, more than one-third of diabetes-related fatalities occur among adults under the age of sixty [6]. In 2024, an estimated 589 million adults aged 20–79 years globally were living with diabetes, accounting for approximately 1 in 9 adults, with projections indicating a rise to 853 million (one in eight adults) by 2050. Notably, 81% of these individuals reside in low- and middle-income countries, underscoring significant global health disparities [7]. This growing prevalence calls for urgent public health interventions and sustainable policy frameworks targeting diabetes prevention and management [4]. Over the last several decades, India has seen a tremendous increase in diabetes prevalence, establishing it as the world’s diabetes capital. According to the International Diabetes Federation (IDF), there were roughly 77 million diabetics in India in 2019, expected to climb to 134 million by 2045 [8]. This fast growth is ascribed to a variety of causes, including urbanization, sedentary lifestyles, poor eating habits, and genetic susceptibility [9]. One of the most significant obstacles to controlling diabetes in India is a lack of information and education about the condition [10]. Many people stay undetected or receive poor care, resulting in serious problems, such as cardiovascular diseases, neuropathy, and retinopathy [11]. Rural areas often lack adequate healthcare infrastructure and are in need of public health campaigns, improved access to services, and policies promoting healthier lifestyles [10].

Understanding the variables that influence malnutrition, eating habits, and health perceptions in diabetes patients is critical for developing successful therapies. Self-efficacy mediates the relationship between pre-motivational cognitions and engagement in health behaviors, emphasizing the significance of psychological elements in health behavior change. Nutrition knowledge substantially impacts dietary choices, highlighting the necessity for educational initiatives [12]. Dietary knowledge plays a critical role in managing health outcomes, particularly for individuals with chronic conditions such as diabetes. Previous studies have highlighted a link between a lack of nutritional knowledge and an increased risk of mild household food insecurity [13], while improved dietary knowledge can significantly influence food choices and behaviors, enhancing overall health [14]. Research by Qudah [15] indicated that individuals with diabetes who possess better nutritional knowledge are more likely to adhere to balanced meals, reducing diabetes-related comorbidities. Furthermore, community-based educational initiatives have been shown to increase nutritional awareness and improve diabetes management [16]. Nutritional quality, defined by balanced nutrient intake, is essential for reducing malnutrition, boosting immunity, and mitigating chronic disease risks [17], with high-quality diets linked to better glycemic control and fewer diabetic complications. Conversely, poor nutrition, particularly diets high in processed foods and deficient in key nutrients, exacerbates health issues, including stunted growth and weakened immunity [18]. Dietitian-led interventions have proven effective in managing diabetes, with studies indicating that patients receiving specialized dietary care show improved HbA1c levels and metabolic profiles [19,20]. Additionally, lifestyle interventions that incorporate nutrition, physical activity, and behavioral support are critical to improving health outcomes, including weight management and glycemic control [21,22]. However, the cost of such interventions remains a barrier, particularly in low-income populations, where economic factors significantly influence the willingness to pay for nutritional services [23,24]. Malnutrition, especially in low- and middle-income countries, is a major health concern, contributing to conditions such as secondary diabetes and pancreatitis [25], highlighting the importance of nutritional assessments in diabetes care. Additionally, the growing influence of mall nutrition—offering both healthy and unhealthy food options—affects the dietary habits of diabetics, necessitating public health initiatives to promote healthier food choices in these settings. A balanced diet, rich in whole grains, lean proteins, and vegetables, is associated with better glycemic control and fewer complications, while diets high in processed foods exacerbate insulin resistance and cardiovascular issues [26]. Psychological perceptions of food choices also play a role, as positive dietary perceptions are linked to better health outcomes, while negative perceptions may hinder compliance and worsen health [27,28]. These factors collectively underline the importance of tailored dietary interventions that address both nutritional and psychological needs in diabetes management. Strategic planning, informed by variables influencing healthy eating behaviors, is critical for improving dietary habits. Targeted communication tactics can be used in social marketing to influence behavior change. Perceived health threats and product knowledge positively affect eating patterns, suggesting that enhanced health awareness and knowledge can reduce poor dietary habits [29]. Addressing these factors is important for improving diabetes management in India.

Despite significant research, the association between malnutrition, dietary behaviors, and health perceptions in diabetes patients remains underexplored, indicating a need for more mediation and moderation studies to improve health outcomes [30,31]. By filling in this knowledge gap and influencing healthcare policies and practices in India, this study seeks to close the knowledge gap. It poses the research question, “How do food habits and malnutrition affect health perception among diabetic patients in India, and what are the mediating and moderating factors in this relationship?” To resolve this research question, the present study investigates the impact of malnutrition and dietary habits on diabetes patients’ health perceptions in India, as well as the mediating and moderating variables influencing this connection. The study’s goal is to give evidence-based recommendations for healthcare interventions and policy through a complete mediation and moderation analysis. These guidelines can help governments and healthcare practitioners design focused dietary programs and health education campaigns to improve diabetes patients’ quality of life.

## 2. Conceptual Framework

The theoretical framework for the study titled “Modeling Factors Influencing Malnutrition, Food Habits, and Health Perception among Diabetic Patients in India: Mediation and Moderation Analysis” is grounded in the Health Belief Model (HBM) and social cognitive theory (SCT), which offer robust explanations for health-related behavior and perception. The HBM postulates that individuals’ health behaviors are shaped by perceived susceptibility, perceived severity, perceived benefits, and barriers to action, all of which directly influence dietary behavior and health perception [32]. In diabetes, malnutrition stems from food insecurity and poor dietary habits shaped by culture, economics, and awareness. Social cognitive theory highlights self-efficacy, noting that dietary knowledge, dietitian-led and lifestyle interventions, and accessibility shape food behaviors [33,34,35,36]. These food habits, in turn, affect patients’ health perceptions and management of diabetes. The mediation analysis explores how food habits mediate the relationship between socioeconomic factors and health outcomes, while the moderation analysis examines how food habits may moderate these relationships. For instance, studies have shown that socioeconomic status significantly impacts dietary choices and health outcomes in diabetic patients. The research work of [37] highlights the significant role of cultural dietary practices in shaping malnutrition patterns in India, emphasizing the need for a comprehensive approach that considers sociocultural and economic contexts, as well as targeted interventions for improving health perceptions among diabetic patients.

## 3. Materials and Methods

This methodological framework ensures a structured and rigorous approach to exploring the interplay between food habits, malnutrition, and health perception among diabetic patients.

### 3.1. Data Source

The present research is based on primary and secondary data. The secondary data were collected from different published as well as unpublished sources, like research papers, books, magazines, and internet resources. The primary data were collected by designing a survey instrument. The study adopted a quantitative research approach using a cross-sectional survey design to investigate the effects of food habits and malnutrition on health perception among diabetic patients in India. The research incorporated mediation and moderation analysis to examine the pathways and interactive effects of key variables.

### 3.2. Target Population Inclusion Criteria

The target population for the study comprised adult diabetic patients residing in urban and semi-urban areas of North and Central India. Eligible participants were required to be between 25 and 65 years of age, representing a diverse demographic segment managing diabetes. Both type 1 and type 2 diabetic patients were included to ensure a comprehensive assessment of the influence of food habits and malnutrition across varying diabetic conditions. Participants must have had a confirmed diagnosis of diabetes for a minimum duration of one year and could be undergoing treatment through oral medications, insulin therapy, or a combination of both. Furthermore, individuals were required to be literate and capable of understanding the survey questionnaire, which was administered in regional languages or English. Informed consent was a prerequisite for participation.

### 3.3. Exclusion Criteria

The study excluded pregnant or nursing women, individuals with gestational diabetes or secondary forms of diabetes, and patients with severe comorbid conditions, such as advanced kidney or liver diseases that might independently influence health perception. Additionally, individuals who exhibited cognitive or physical limitations that prevented them from independently completing the survey were not considered for inclusion in the study.

### 3.4. Data Collection Instruments and Questionnaires

Using instruments and variables created based on earlier research, the study used validated questionnaires to evaluate the construct of interest. By classifying responses into good and poor eating habits, the Food Habits Questionnaire (FHQ) assesses dietary patterns. Higher scores are given to healthier dietary practices. Using BMI, inadvertent weight loss, and acute disease consequences, the Malnutrition Universal Screening Tool (MUST) evaluates malnutrition risk, where scores represent low, medium, or high risk. Using a Likert scale, the Health Perception Questionnaire (HPQ) gauges self-reported perceptions of one’s physical and mental health, where higher scores indicate greater health. While moderation analysis looks at the impact of demographic characteristics like age, gender, and type of diabetes, mediation analysis looks at malnutrition as an intermediary variable between eating habits and health perception. The questionnaires were meticulously designed to cover dimensions such as dietary knowledge, nutritional quality, dietitian-led and lifestyle interventions, and cost and willingness, drawing on the research in [16,25,37], all of which contributed to the development of eating habits and health outcome constructs.

### 3.5. Sampling Strategy

Data were collected online as well as offline, contacting the patient by visiting their location as well as contacting them online with Google Docs through various social media sites, like Facebook, Research Gate, LinkedIn, Instagram, etc. The questionnaire was initially circulated to 500 respondents, and it was further requested to be passed on by them. The research received 550 responses. After editing, 503 responses were found fit and taken for the study.

### 3.6. Common Method Bias

To assess the presence of common method bias, Harman’s single-factor test was employed. This involved conducting an exploratory factor analysis (EFA) on all measured items to determine whether a single factor emerged or whether one factor accounted for the majority of the variance. The analysis revealed that the first factor accounted for 43.505% of the total variance, which is below the threshold of 50%. This indicates that common method bias was not a significant concern in this study.

### 3.7. Reliability and Validity

The study ensured reliability and validity through the use of established measurement scales and robust statistical procedures. The use of SPSS 22 and SmartPLS 3.0 helped enhance data reliability by systematically analyzing and refining the measurement model. The questionnaire was validated by academics and subject experts and pilot-tested on 30 patients, resulting in a reliability score of 0.916 (Cronbach’s α = 0.70), indicating its eligibility for future investigation. Ethical guidelines, such as informed consent, confidentiality, and voluntary participation, were followed with prior approval obtained before data collection.

### 3.8. Data Analysis

Quantitative data were analyzed using structural equation modeling (SEM) to evaluate both direct and indirect relationships among the variables. Mediation and moderation analyses were conducted to explore the underlying mechanisms and contextual influences between food habits and health perception. The dual use of SPSS 22 and SmartPLS 3.0 facilitated comprehensive data analysis and ensured the robustness of findings by accommodating both reflective and formative constructs.

## 4. Results

The information related to the demographic characteristics of the respondents revealed that the largest group comprised those aged 26–35 years (23.7%), followed closely by 18–25 years (22.7%) and 46–55 years (20.1%). The gender distribution showed a higher proportion of males (61.8%) compared to females (38.2%). Marital status indicated a predominance of married individuals (61.0%), with unmarried respondents at 32.6% and those divorced or separated at 6.4%. Occupationally, unorganized workers formed the largest group (39.8%), followed by those in farming (23.7%) and service sector employees (15.5%). Educationally, a significant portion had attained graduation (25.4%), with notable proportions having technical qualifications (20.7%) and intermediate education (15.7%). Monthly income levels showed that a large segment earned up to Rs. 1500 PM (44.9%), with smaller groups in higher income brackets. Regarding the duration of suffering from diabetes, the majority had been afflicted for more than 15 years (38.8%), with notable groups experiencing it for 11–15 years (33.4%) and 6–10 years (13.9%). This comprehensive demographic overview provided valuable insights into the varied backgrounds and health statuses of the respondents. Table 1 indicates the demographic characteristics of the respondents.

The descriptive statistics in Table 2 highlight the factors influencing malnutrition, food habits, and health perception among diabetic patients. The construct of dietary knowledge (DK) showed a mean of 4.071 with a standard deviation of 0.657 and variance of 0.431, indicating that patients generally had good nutrition knowledge, understanding the importance of balancing macronutrients (mean = 4.127, SD = 0.835) and recognizing beneficial foods for diabetes management (mean = 3.831, SD = 0.802). Nutritional quality reflected a mean of 4.158, suggesting high dietary quality with moderate variability (SD = 0.675, variance = 0.456). The construct of dietitian-led intervention had a mean of 3.867 (SD = 0.773, variance = 0.598), indicating positive perceptions toward dietitian-led health interventions, though the responses for smart technology applications within this construct showed greater variability. Lifestyle interventions (mean = 3.903, SD = 0.749, variance = 0.561) highlighted that regular physical activity and adopting a healthy lifestyle are essential for diabetes management. The cost and willingness to pay construct revealed a mean of 3.715, with some challenges noted in affording healthy food (SD = 0.724, variance = 0.524). The malnutrition construct had a mean of 3.793, reflecting awareness of nutritional information and availability of healthy food options (SD = 0.662, variance = 0.438). Food habits showed a high mean of 4.0282, indicating regular balanced meals and healthy snacking habits (SD = 0.642, variance = 0.412). Finally, perceived health outcome had a mean of 3.9105, underscoring the perceived positive health impacts of healthy eating habits on diabetes management (SD = 0.653, variance = 0.426). These findings underscore the importance of dietary knowledge, quality nutrition, and lifestyle interventions in managing diabetes effectively.

A measurement model in the context of nursing and health professions refers to the evaluation and validation of the relationship between latent variables and observed variables. In the analysis of factors influencing malnutrition, food habits, and health perception among diabetic patients using partial least squares structural equation modeling (PLS-SEM), the measurement model demonstrated varying degrees of construct reliability and validity. Construct reliability, assessed via Cronbach’s alpha and composite reliability (rho_a and rho_c), indicated acceptable to high internal consistency across constructs, with values ranging from 0.805 to 0.931, all surpassing the standard threshold of 0.70, thus confirming reliable measurement (see Table 3). The average variance extracted (AVE) values, ranging from 0.509 to 0.782, exceeded the acceptable threshold of 0.50, ensuring adequate convergent validity. Collinearity statistics (VIF) showed acceptable levels, with VIF values below the critical threshold of 5, indicating no severe multicollinearity issues. The F-square effect sizes, ranging from 0.023 to 0.139, suggested varying levels of impact, with only cost and willingness to pay and dietitian-led intervention demonstrating moderate effect sizes, indicating their substantial role in influencing the constructs. Overall, the model exhibited robust reliability and validity, aligning with established thresholds, thereby reinforcing the validity of the findings and their implications for understanding diabetic patients’ nutrition and health perceptions.

The discriminant validity of the constructs in the study was assessed using two methods: the Fornell–Larcker criterion and the heterotrait–monotrait ratio (HTMT). According to Table 4, the Fornell–Larcker criterion revealed that the square root of the AVE for each construct exceeded the correlations with other constructs, indicating satisfactory discriminant validity. Specifically, nutritional quality and perceived health outcome demonstrated strong discriminant validity, with AVE values of 0.884 and 0.850, respectively, which were significantly higher than their correlations with other constructs, confirming they were distinct from others.

Table 5 provides the HTMT ratios, where values below the threshold of 0.85 suggest adequate discriminant validity. Constructs such as nutritional quality exhibited a low HTMT ratio (0.239) with other constructs, further supporting its distinctiveness. However, constructs like food habits and malnutrition showed higher HTMT ratios (up to 0.957), suggesting closer interrelationships that may need further investigation to ensure clear conceptual boundaries.

Table 6 presents the predictive performance of the model, with R-squared (R^2^) values indicating the proportion of variance explained by the predictors. The R^2^ values for food habits (0.676) and malnutrition (0.733) were relatively high, suggesting that the model explained a significant portion of the variance in these constructs. The Q^2^ values, which were above zero for food habits (0.750) and malnutrition (0.722), supported the model’s predictive relevance according to the Stone–Geisser criterion. Conversely, the perceived health outcome had a lower R^2^ (0.480) and Q^2^ (0.526), indicating a less robust model fit for this outcome. The root mean square error (RMSE) and mean absolute error (MAE) values for all constructs reflected reasonable model accuracy, with food habits and malnutrition demonstrating lower RMSE values (0.503 and 0.530, respectively) compared to perceived health outcome (0.692). These findings suggest that while the model performed well in predicting food habits and malnutrition, its predictive capability for perceived health outcomes could be improved.

The model fit indices presented in Table 7 offer insights into the overall model performance. The saturated model showed a standardized root mean square residual (SRMSR) of 0.075 and other fit indices, such as d_ULS (4.899) and d_G (3.011), indicating a good fit relative to the data. However, the estimated model’s SRMSR of 0.093 slightly exceeded the common threshold of 0.08, suggesting a less optimal fit. The d_ULS (7.461) and d_G (3.262) values, although higher than the saturated model, still fell within an acceptable range, yet they indicated some misfit. The Chi-square statistic for the estimated model (7427.799) was higher compared to the saturated model (7116.940), reflecting a larger discrepancy between the model and the observed data. Additionally, the Normed Fit Index (NFI) decreased from 0.663 in the saturated model to 0.648 in the estimated model, signaling a decline in model fit. These results collectively suggest that while the model generally fit the data, there were areas for potential improvement in its specification.

The SmartPLS outcome in Table 8 presents the results of structural model and hypothesis testing, illustrating path coefficients, standard deviations (SDs), T-test values, and *p*-values for various paths. The coefficient from dietary knowledge to malnutrition (β = 0.126, *p* = 0.006) was significant, indicating that dietary knowledge positively influenced malnutrition. Similarly, the paths from dietitian-led intervention to malnutrition (β = 0.339, *p* < 0.001) and cost and willingness to pay for malnutrition (β = 0.340, *p* < 0.001) were significant, showing strong positive impacts. Lifestyle interventions also significantly affected malnutrition (β = 0.133, *p* = 0.011). However, nutritional quality did not significantly impact malnutrition (β = 0.050, *p* = 0.140).

Malnutrition significantly influenced perceived health outcome (β = 0.368, *p* < 0.001) and food habits (β = 0.822, *p* < 0.001), with food habits also affecting perceived health outcome (β = 0.313, *p* < 0.001). The interaction effect of food habits and malnutrition on perceived health outcomes was negative and significant (β = −0.051, *p* = 0.007), indicating a moderation effect. Additionally, the indirect path from malnutrition to perceived health outcome through food habits was significant (β = 0.258, *p* < 0.001), demonstrating mediation. These results collectively highlighted the crucial roles of dietary knowledge, dietitian-led interventions, cost factors, and lifestyle interventions in addressing malnutrition and improving health outcomes, with significant mediation and moderation effects observed in the model.

## 5. Discussion

The current study examined the influence of food habits and malnutrition on perceived health outcomes among diabetic patients in India, focusing on both direct and indirect pathways, and identified key mediating and moderating variables in this complex relationship. The findings affirmed the centrality of malnutrition and dietary behaviors in shaping health perceptions among individuals with diabetes, a group that remains particularly vulnerable due to chronic metabolic dysregulation and heightened sensitivity to dietary inadequacies. The study emphasized the importance of dietary knowledge in treating malnutrition in diabetes patients. Educating these persons about good dietary behaviors is vital for improving their nutritional status, as demonstrated by past studies that link enhanced dietary knowledge with better dietary choices and overall nutritional well-being for those with chronic conditions like diabetes [38]. Dietary knowledge, dietitian-led interventions, cost and willingness to pay, and lifestyle interventions emerged as significant predictors of malnutrition. These factors align with previous research emphasizing the importance of dietary education and professional guidance in managing nutritional deficiencies among diabetics [39].

The analysis indicated that malnutrition significantly influenced food habits, which subsequently shaped health perceptions among diabetic patients, corroborating global findings that poor nutrition worsens diabetes outcomes and reduces perceived well-being [40]. The mediating role of food habits between malnutrition and health perception supports earlier studies emphasizing diet quality as vital to health-related quality of life in chronic illnesses [41]. Dietitian-led interventions play a crucial role in combating malnutrition, underscoring the importance of professional guidance in diabetes care [42]. Although our study found no direct link between nutritional quality and malnutrition, prior research affirmed its significance in dietary management [43]. Furthermore, the financial difficulties that diabetes patients have in acquiring healthy meals have a substantial impact on malnutrition. This is consistent with previous research indicating that economic restrictions might harm food quality, resulting in malnutrition [44]. Lifestyle therapies, such as physical exercise and healthy eating habits, are also important in controlling malnutrition among diabetes patients. Lifestyle adjustments have regularly been proven in research to enhance diabetes patients’ health outcomes [39,45].

Interestingly, the study indicated that nutritional quality alone had no significant influence on malnutrition, indicating that factors such as dietary awareness, professional advice, and economic ability may play more direct roles in resolving malnutrition in diabetes patients. This finding highlights the need for more studies to investigate the function of dietary quality in this setting more thoroughly. The study also emphasized the importance of malnutrition on perceived health outcomes and dietary behaviors. Malnutrition not only had a direct impact on health perceptions, but it also changed dietary patterns, which in turn influenced general health. These findings are consistent with previous studies indicating malnutrition’s larger ramifications for both physical and mental health [46,47].

The substantial positive link between malnutrition and perceived health outcomes emphasized the negative consequences of low nutritional status on overall health perceptions, which supports previous research [48]. Furthermore, the significant mediation effect of food habits on the relationship between malnutrition and health perception highlighted the complex interplay between dietary behaviors and health outcomes, which is consistent with findings that emphasized the holistic approach required for effective diabetes management [49]. A novel contribution of this study is the moderating role of food habits in the relationship between malnutrition and perceived health outcomes. The negative interaction suggested that when poor food habits coexist with malnutrition, the perceived health deteriorates more sharply. This finding challenges some earlier works, which emphasized either malnutrition or dietary behavior independently [50], and it supports the growing consensus that a dual-burden model—where both nutrition and behavior coalesce—provides a more accurate depiction of health dynamics in chronic disease populations. The observed moderation effect implied that the detrimental impact of malnutrition on health perceptions might be amplified by poor eating habits, highlighting the importance of comprehensive therapies that target both nutrition and lifestyle determinants. These findings highlighted the urgent need for tailored interventions that focus on nutritional education, professional supervision, and economic considerations to reduce malnutrition and improve health outcomes for diabetes patients in India.

## 6. Theoretical and Managerial Implications

This research deepened the theoretical understanding of factors influencing malnutrition and health perceptions among diabetic patients in India. It found that dietary knowledge, dietitian-led interventions, and willingness to pay positively impacted malnutrition, consistent with literature emphasizing education and professional guidance. The study also underscored the importance of lifestyle interventions, advocating for holistic approaches that combine physical activity with behavioral changes. The lack of significant effect from nutritional quality alone highlighted the need for integrated educational strategies. Additionally, the mediation of food habits on malnutrition and perceived health outcomes emphasized the critical role of dietary behavior in health perception. The observed moderation effect suggested that the relationship between malnutrition and food habits is complex and context-dependent, warranting further theoretical investigation.

## 7. Managerial and Policy Implications

From a managerial standpoint, the findings provided valuable insights for enhancing the nutritional health of diabetic patients in India. Emphasizing dietary education through structured programs can effectively reduce malnutrition. Healthcare providers should focus on dietitian-led interventions, which are crucial for effective malnutrition management. Policymakers need to address economic barriers and advocate for affordable nutritional programs. Integrating lifestyle interventions, such as physical activity, into patient care is essential for a holistic approach to diabetes management.

The results of the study underlined important policy implications for handling the related issues of eating habits and malnutrition in forming health perception among diabetic patients in India. The study highlighted the importance of addressing eating habits and malnutrition in shaping health perception among diabetic patients in India. Dietitian-led lifestyle changes significantly impacted malnutrition levels and economic factors like cost and willingness to pay. Malnutrition not only mediated the link between eating behaviors and perceived health but also moderated it. The findings emphasized the need for integrated healthcare policies that prioritize nutritional education, broaden access to professional dietary counseling, and consider affordability issues. These strategies can reduce malnutrition, promote better eating habits, and improve health attitudes and quality of life for diabetic patients.

## 8. Limitations and Future Scope of the Study

The study “Modeling Factors Influencing Malnutrition, Food Habits, and Health Perception among Diabetic Patients in India” provided valuable insights into the multifaceted relationships between dietary knowledge, dietitian-led interventions, cost factors, lifestyle interventions, and their impacts on malnutrition, food habits, and perceived health outcomes. However, several limitations should be acknowledged. First, the study’s cross-sectional design restricted the ability to draw causal inferences. Longitudinal studies could provide more definitive evidence of causality. Second, the reliance on self-reported data might introduce response biases, affecting the accuracy of the findings. Future research could incorporate objective measures of dietary intake and health outcomes. Third, the study focused on a specific demographic group, which may limit the generalizability of the results to other populations. Expanding the research to diverse demographic groups would enhance its applicability. Additionally, the impact of sociocultural factors on dietary habits and health perceptions was not deeply explored and warrants further investigation. While the study adjusted for sociodemographic confounders like education, income, occupation, and age, their non-inclusion as standalone variables represents a limitation, as such factors could exert indirect effects on the relationships under study. Future research should incorporate these variables to refine the model’s predictive power and better capture their nuanced impact on dietary habits, malnutrition, and health perception. Future studies could also explore the role of emerging technologies, such as mobile health applications and telemedicine, in managing malnutrition and promoting healthy food habits among diabetic patients. Finally, while the mediation and moderation effects observed provide a robust understanding of the interrelationships among variables, further exploration using more complex statistical models could offer deeper insights into these dynamics.

## 9. Conclusions

The study provided critical insights into the determinants of malnutrition and their impacts on health perceptions among diabetic patients. The findings highlighted the significant roles of dietary knowledge, dietitian-led interventions, and cost factors, alongside lifestyle interventions in mitigating malnutrition. Specifically, dietary knowledge, dietitian-led interventions, and willingness to pay are strong positive influencers of malnutrition management. Although nutritional quality was not a significant factor, lifestyle interventions were crucial in addressing malnutrition. Malnutrition was found to significantly influence both perceived health outcomes and food habits, with food habits also impacting health perceptions. The study revealed a significant negative interaction effect between food habits and malnutrition on health outcomes, indicating a moderation effect, while the mediation analysis underscored the indirect impact of malnutrition on health outcomes through food habits. These results corroborate previous research, emphasizing the multifaceted approach needed to tackle malnutrition in diabetic patients and improve their overall health perceptions through comprehensive dietary and lifestyle strategies, supported by professional guidance and economic considerations.

## Figures and Tables

**Table 1:** Demographic characteristics of the respondents (*N* = 503).

Characteristics	Description	Frequency	Percent
Age Categories	Up to 18 years	49	9.7
	18–25 years	114	22.7
	26–35 years	119	23.7
	36–45 years	87	17.3
	46–55 years	101	20.1
	56–65 years	33	6.6
Gender	Male	311	61.8
	Female	192	38.2
Marital Status	Married	307	61.0
	Unmarried	164	32.6
	Divorce/Separated	32	6.4
Occupation	Farming	119	23.7
	Business	34	6.8
	Service	78	15.5
	Unorganized workers	200	39.8
	Housewife	47	9.3
	Others	25	5.0
Education Qualification	Elementary education	78	15.5
	Up to Matric	43	8.5
	Intermediate	79	15.7
	Graduation	128	25.4
	Post graduation	41	8.2
	Technical qualification (ITI/Diploma/Engineering Degree)	104	20.7
	Other qualification	30	6.0
Monthly Income	Up to Rs. 1500 PM	226	44.9
	Rs. 1501 to Rs. 2500 PM	82	16.3
	Rs. 25,001 to Rs. 50,000 PM	82	16.3
	Rs. 50,001 to Rs. 100,000 PM	68	13.5
	Above Rs. 100,000 PM	45	8.9
Diabetes Onset	Since 3 years	43	8.5
	3–5 years	27	5.4
	6–10 years	70	13.9
	11–15 years	168	33.4
	More than 15 years	195	38.8

**Table 2:** Factors influencing malnutrition, food habits, and health perception among diabetic patients: descriptive statistics (N = 503).

Construct and Measurement Variable	Mean	SD	Variance
Dietary Knowledge (DK)	4.071	0.657	0.431
I am knowledgeable about the nutritional content of the foods I eat.	3.950	0.818	0.669
I understand the importance of balancing carbohydrates, proteins, and fats in my diet.	4.127	0.835	0.697
I can identify foods that are beneficial for managing my diabetes.	3.831	0.802	0.643
I am aware of the recommended daily intake of vitamins and minerals.	4.241	0.765	0.585
I know how to read and understand food labels.	4.207	0.715	0.511
Nutritional Quality	4.158	0.675	0.456
I consume a diet rich in fruits and vegetables.	4.044	0.777	0.604
I eat whole grains instead of refined grains.	4.078	0.835	0.697
I avoid foods high in added sugars and unhealthy fats.	4.109	0.790	0.623
I ensure my meals are balanced and nutritious.	4.280	0.719	0.517
I limit my intake of processed and fast foods.	4.278	0.704	0.496
Dietitian-Led Intervention	3.867	0.773	0.598
I find dietitian-led interventions helpful in managing my diet.	3.968	0.867	0.752
I regularly consult a dietitian for dietary advice.	3.704	1.076	1.157
Dietitian-led interventions have improved my knowledge about diabetes management.	3.813	0.964	0.929
I feel more confident in my dietary choices after consulting a dietitian.	4.010	0.848	0.719
Dietitian-led interventions have positively impacted my health.	3.841	0.948	0.899
Lifestyle Interventions	3.903	0.749	0.561
I engage in regular physical activity to manage my diabetes.	3.915	1.037	1.074
I have adopted a healthy lifestyle to improve my overall well-being.	3.787	0.934	0.873
Lifestyle interventions have helped me manage my diabetes effectively.	3.815	0.901	0.812
I prioritize getting enough sleep and managing stress.	3.946	1.098	1.206
Lifestyle interventions are essential for my diabetes management.	4.050	0.912	0.832
Cost and Willingness to Pay	3.715	0.724	0.524
I am willing to pay for high-quality, nutritious food.	4.008	0.955	0.912
I find it challenging to afford healthy food options.	3.885	1.009	1.019
I am willing to invest in dietitian-led interventions.	3.809	0.942	0.888
The cost of healthy food is a barrier to my dietary management.	3.773	0.906	0.821
I believe the benefits of a healthy diet outweigh the costs.	3.097	1.026	1.052
Malnutrition	3.793	0.662	0.438
I am aware of the nutritional information provided at food outlets.	3.670	1.017	1.034
I understand how to read and interpret nutritional labels at food outlets.	3.624	0.930	0.865
The availability of healthy food options influences my dining choices.	3.565	1.093	1.195
I limit my intake of sugary and high-calorie foods when eating.	3.930	0.871	0.758
My blood sugar levels are better managed when I eat healthy food.	3.767	0.788	0.621
I have experienced weight management benefits from eating healthier options at malls.	4.010	0.867	0.751
My overall diabetes management has improved due to the healthier food choices available at the outlet.	3.984	0.951	0.904
Food Habits	4.028	0.642	**0.412**
I regularly eat three balanced meals a day.	3.897	0.776	0.603
I snack on healthy foods between meals.	4.022	0.740	0.547
My dietary habits positively influence my diabetes management.	3.988	0.720	0.518
I perceive my overall health to be good.	4.086	0.751	0.564
I believe that my food habits are crucial for my health.	4.149	0.665	0.442
Perceived Health Outcome	3.911	0.653	0.426
My blood sugar levels are better managed when I eat healthy food available at mall outlets.	3.845	1.041	1.083
I have experienced weight management benefits from eating healthier options at malls.	3.950	0.899	0.808
My overall diabetes management has improved due to the healthier food choices.	3.885	0.862	0.744
I have experienced weight changes due to my eating habits.	3.962	0.750	0.562

**Table 3:** Construct reliability and validity, collinearity, and F-square test statistics.

	Cronbach’s Alpha	Composite Reliability (rho_a)	Composite Reliability (rho_c)	Average Variance Extracted (AVE)	Collinearity Matrix	F-Square
Cost and _Willingness to Pay	0.805	0.816	0.866	0.566	3.410	0.127
Dietary_ Knowledge	0.893	0.904	0.923	0.706	2.057	0.029
Dietitian-Led _Intervention	0.883	0.892	0.915	0.686	3.098	0.139
Food Habits	0.928	0.930	0.945	0.776	3.127	0.060
Lifestyle _Interventions	0.823	0.828	0.876	0.587	2.928	0.023
Malnutrition	0.837	0.855	0.878	0.509	1.000	2.083
Nutritional_ Quality	0.931	0.963	0.947	0.782	1.066	0.082
Perceived Health Outcome	0.868	0.918	0.911	0.722		0.009

**Table 4:** Discriminant validity: Fornell–Larcker criterion.

	Cost and _Willingness to Pay	Dietary_ Knowledge	Dietitian-Led _Intervention	Food Habits	Lifestyle _Interventions	Malnutrition	Nutritional_ Quality	Perceived Health Outcome
Cost and _Willingness to Pay	0.752							
Dietary_ Knowledge	0.633	0.841						
Dietitian-Led _Intervention	0.797	0.627	0.828					
Food Habits	0.803	0.655	0.870	0.881				
Lifestyle _Interventions	0.752	0.685	0.720	0.746	0.766			
Malnutrition	0.801	0.654	0.795	0.822	0.730	0.714		
**Nutritional_ Quality**	0.222	0.197	0.207	0.226	0.238	0.252	0.884	
Perceived Health Outcome	0.680	0.642	0.654	0.652	0.756	0.663	0.145	0.850

**Table 5:** Discriminant validity: heterotrait–monotrait ratio (HTMT)—matrix.

	Cost and _Willingness to Pay	Dietary_ Knowledge	Dietitian-Led _Intervention	Food Habits	Lifestyle _Interventions	Malnutrition	Nutritional_ Quality	Perceived Health Outcome	Food Habits × Malnutrition
Cost and _Willingness to Pay									
Dietary_ Knowledge	0.741								
Dietitian-Led _Intervention	0.842	0.703							
Food Habits	0.726	0.707	0.856						
Lifestyle _Interventions	0.826	0.791	0.846	0.848					
Malnutrition	0.957	0.749	0.804	0.808	0.873				
Nutritional_ Quality	0.239	0.209	0.212	0.228	0.258	0.273			
Perceived Health Outcome	0.803	0.706	0.737	0.700	0.871	0.753	0.138		
Food Habits × Malnutrition	0.448	0.185	0.374	0.451	0.505	0.490	0.150	0.407	

**Table 6:** R-squared, Q^2^ predict, RMSE, and MAE: an overview.

	R-Square	R-Square Adjusted	Q^2^ Predict	RMSE	MAE
Food Habits	0.676	0.675	0.750	0.503	0.391
Malnutrition	0.733	0.731	0.722	0.530	0.400
Perceived Health Outcome	0.480	0.477	0.526	0.692	0.540

**Table 7:** Model fit summary.

	Saturated Model	Estimated Model
SRMSR	0.075	0.093
d_ULS	4.899	7.461
d_G	3.011	3.262
Chi-square	7116.940	7427.799
NFI	0.663	0.648

**Table 8:** Structural model and hypothesis testing: path coefficients, mean, SD, T-test values, and *p*-values.

	Path Coefficient (β)	Standard Deviation (SD)	T Statistics (|O/SD|)	*p*-Values
Dietary_ Knowledge -> Malnutrition	0.126	0.045	2.761	0.006
Nutritional_ Quality -> Malnutrition	0.050	0.034	1.475	0.140
Dietitian-Led _Intervention -> Malnutrition	0.339	0.050	6.822	0.000
Lifestyle _Interventions -> Malnutrition	0.133	0.052	2.556	0.011
Cost and _Willingness to Pay -> Malnutrition	0.340	0.058	5.860	0.000
Malnutrition -> Perceived Health Outcome	0.368	0.070	5.298	0.000
Malnutrition -> Food Habits	0.822	0.016	52.311	0.000
Food Habits -> Perceived Health Outcome	0.313	0.062	5.081	0.000
Food Habits × Malnutrition -> Perceived Health Outcome	−0.051	0.019	2.722	0.007
Malnutrition -> Food Habits -> Perceived Health Outcome	0.258	0.051	5.080	0.000

## Data Availability

The data presented in this study are available upon request from the first author due to privacy.
